# ROOT HAIR DEFECTIVE SIX‐LIKE4 (RSL4) promotes root hair elongation by transcriptionally regulating the expression of genes required for cell growth

**DOI:** 10.1111/nph.14095

**Published:** 2016-07-25

**Authors:** Priya Vijayakumar, Sourav Datta, Liam Dolan

**Affiliations:** ^1^ Department of Plant SciencesUniversity of OxfordOxfordOX1 3RBUK

**Keywords:** Arabidopsis cell growth, basic helix‐loop‐helix transcription factor, CALCIUM‐DEPENDENT PROTEIN KINASE11 (CPK11), EXOCSYT SUBUNIT 70A1 (EXO70A1), PEROXIDASE7 (PRX7), root hair, ROOT HAIR DEFECTIVE SIX‐LIKE4 (RSL4), SUPPRESSOR OF ACTIN (SAC1)

## Abstract

- ROOT HAIR DEFECTIVE SIX‐LIKE4 (RSL4) is necessary and sufficient for root hair elongation in *Arabidopsis thaliana*. Root hair length is determined by the duration for which RSL4 protein is present in the developing root hair. The aim of this research was to identify genes regulated by RSL4 that affect root hair growth.
- To identify genes regulated by RSL4, we identified genes whose expression was elevated by induction of RSL4 activity in the presence of an inhibitor of translation.
- Thirty‐four genes were identified as putative targets of RSL transcriptional regulation, and the results suggest that the activities of SUPPRESSOR OF ACTIN (SAC1), EXOCSYT SUBUNIT 70A1 (EXO70A1), PEROXIDASE7 (PRX7) and CALCIUM‐DEPENDENT PROTEIN KINASE11 (CPK11) are required for root hair elongation.
- These data indicate that RSL4 controls cell growth by controlling the expression of genes encoding proteins involved in cell signalling, cell wall modification and secretion.

## Introduction

Root hairs are tubular projections that develop from the outermost layer of roots (epidermis or periderm). They can constitute up to *c*. 50% of the surface area of the root and extend the absorptive surface of the root into the surrounding soil, facilitating the uptake of nutrients, such as phosphate, with limited mobility in the soil. Growth is restricted to the tip of the root hair; no growth occurs along the shanks. New cell surface is generated though the localized secretion of new membrane and cell wall components from the endomembrane system to a spatially restricted area at the root hair tip (Carol & Dolan, 2002; Datta *et al*., 2011; Balcerowicz *et al*., 2015; Salazar‐Henao & Schmidt, 2016). Growth at the tip is coordinated by a number of activities including Ca^2+^, pH and reactive oxygen oscillations at the tip (Ishida *et al*., 2008; Datta *et al*., 2011; Salazar‐Henao & Schmidt, 2016).

The growth of root hairs is determined by the interaction of a plethora of external environmental signals with internal growth regulators. Phosphate availability is the best‐characterized external factor that influences root hair length (Bates & Lynch, 2000; Lopez‐Bucio *et al*., 2003; Zhang *et al*., 2003; Lee & Cho, 2013; Niu *et al*., 2013). In most species, root hairs are longer in roots grown in low‐phosphate conditions than when grown in replete phosphate conditions. This adaptive response to low phosphate availability increases the capacity for phosphate uptake because longer hairs can mine larger volumes of the soil for phosphate (Brown *et al*., 2013; Lopez‐Arredondo *et al*., 2014). Such phenotypic plasticity is characteristic of plant development and is often under strict transcriptional control (Rubio *et al*., 2001; Jain *et al*., 2012).

The ROOT HAIR DEFECTIVE SIX‐LIKE (RSL) class I basic helix‐loop‐helix (bHLH) transcription factors positively regulate the development of root hairs in angiosperms and rhizoids in liverworts and mosses (Menand *et al*., 2007; Yi *et al*., 2010; Proust *et al*., 2016). *Arabidopsis thaliana* plants deficient in RSL class I activity – *root hair defective six rsl1* double mutants – do not develop root hairs (Menand *et al*., 2007). Similarly, no rhizoids develop in the *rsl1* mutant of the liverwort *Marchantia polymorpha* or the *Pprsl1 Pprsl2* double mutant of the moss *Pyscomitrella patens* (Menand *et al*., 2007; Proust *et al*., 2016). *Arabidopsis thaliana* RSL class I genes act, at least in part, by directly regulating the expression of RSL4, a closely related bHLH transcription factor (Yi *et al*., 2010). RSL4 is necessary for root hair elongation; few short root hairs develop on *rsl4* mutants (Yi *et al*., 2010). Furthermore, RSL4 is also sufficient for root hair growth as constitutive expression of RSL4 results in the constitutive growth of root hairs (Yi *et al*., 2010). During wild‐type development, RSL4 is produced in a pulse at the beginning of root hair development and growth continues until RSL4 is removed from the cell through proteolysis (Datta *et al*., 2015). Mutated forms of RSL4 that are more stable than the wild‐type form programme growth for a longer time than the wild‐type form, leading to the development of longer root hairs than in the wild‐type (Datta *et al*., 2015). This demonstrates the role of RSL4 stability in determining root hair length. Taken together, these data indicate that RSL4 is a potentiator of root hair growth that is produced in a pulse just before growth initiates, and growth ceases once proteolysis removes the last of the RSL4 from the growing root hair.

Low phosphate‐induced root hair elongation is mediated, at least in part, by modulation of RSL4 expression (Svistoonoff *et al*., 2007; Yi *et al*., 2010; Koltai & Kapulnik, 2013; Lopez‐Arredondo *et al*., 2014). Low concentrations of phosphate in the growth media increase the steady‐state levels of RSL4 mRNA (Yi *et al*., 2010). This increase does not occur in mutants that are defective in phosphate sensing, indicating that the increase is a target of the phosphate‐sensing mechanism (Yi *et al*., 2010). The amount of RSL4 synthesized in a pulse just before root hair initiation is higher in low phosphate than in replete phosphate. Because the amount of RSL4 synthesized is greater, the duration of growth is increased, resulting in development of longer root hairs compared with plants grown in replete phosphate where less RSL4 is produced before root hair initiation (Datta *et al*., 2015). These data indicate that RSL4 is a key modulator of root hair growth and the target of pathways that modulate root hair elongation.

To determine how RSL4 controls root hair elongation, we set out to identify genes encoding proteins that are targets of RSL4 transcriptional regulation. Here, we identified 34 putative targets of RSL4 and demonstrated that four of these genes are required for root hair growth and overexpression of two of the genes increases root hair elongation. These data indicate that RSL4 regulates the expression of genes involved in cell signalling, vesicle transport and cell wall modification.

## Materials and Methods

### Plant material and growth conditions

Arabidopsis (*Arabidopsis thaliana*, (L.) Heynh.) ecotype Columbia (Col‐0) was used for all the experiments. The T‐DNA insertion mutant seeds were obtained from the Arabidopsis Biological Resource Center (ABRC) and the Nottingham Arabidopsis Stock Centre (NASC). The Arabidopsis seeds were surface‐sterilized in 70% ethanol for 5 min followed by 5% (v/v) bleach for 10 min and rinsed with sterile distilled water three times. For the T‐DNA insertion lines and the Glucocorticoid Receptor (GR) inducible lines, the seeds were plated on Murashige and Skoog (MS) medium (pH 5.8) supplemented with 1% sucrose (w/v) and 0.5% Phytagel (w/v) (Sigma, UK). For the overexpression studies, at least four independent transgenic lines overexpressing the target genes were examined. In order to observe the root hair phenotype of the overexpression lines, the plants were grown vertically at a 45° angle in half‐strength Johnson medium with 0.5% Phytagel. After a period of stratification at 4°C for 3 d, the plants were grown vertically under continuous illumination at 25°C in the growth chamber. The roots were observed 6–7 d after sowing.

### Generation of GR‐fusion constructs

To generate the N‐terminal GR fusion vector, the multisite gateway system was used. Gateway entry clones were generated using pDONR P4‐P1R containing the promoter plus the 5′ untranslated region (UTR) of the gene, pDONR P2R‐P3 cloned with the coding region of the gene plus 3′ UTR and terminator and the GR domain inserted into pDONR 207 as described (Yi *et al*., 2010). Gateway LR multisite reaction (Gateway LR clonase^ ^reaction mix; Invitrogen) was carried out with pDONR P4‐P1R, pDONR P2R‐P3, pDONR 207‐GR plus the binary destination vector pGWB multisite in order^ ^to generate the GR:RSL4 fusion vector. Primers used to check the GR:RSL4 constructs are listed in Supporting Information Table S1. The binary vector pGWB multisite was a gift from Matt Tomlinson (John Innes Centre, Norwich, UK) and was generated by replacing the R1‐CmR‐ccdB‐R2 cassette of pGWB1 with R4‐CmR‐ccdB‐R3 (pGWB1 was from Tsuyoshi Nakagawa, Shimane University, Japan). This construct was then transformed into the *rsl2 rsl4* mutant background.

### Plant transformation

The *Agrobacterium tumefaciens*‐mediated floral dip method was used to transform all the binary vectors into Arabidopsis (Clough & Bent, 1998). Plants with a few siliques and several immature buds were used for transformation. A volume of 250 ml of *A. tumefaciens* culture incubated at 28°C overnight was spun down and the pellet was re‐suspended in 200 ml of solution containing 5% (w/v) sucrose and 0.05% (v/v) Silwet L‐77. The floral tissues of the Arabidopsis plants were dipped into the bacterial mixture for 30 s, after which the plants were laid down on a tray with wet tissues and covered with cling film overnight. The dipped plants were then returned to the glasshouse.

### Dexamethasone treatment

A 20 mM stock solution of dexamethasone (DEX) in ethanol was used for the treatment. T2 seeds of *GR:RSL4 rsl2 rsl4* plants were first germinated in MS medium containing 25 μg ml^−1^ hygromycin and after 4 d the resistant seedlings were transferred to MS medium without antibiotics. Six‐day‐old seedlings were treated with 20 μM DEX by flooding with 20 ml of MS liquid medium containing 20 μl of DEX (DEX treated) or 20 μl of ethanol (mock treated). After treatment, the root tips were sampled at several time‐points for qRT‐PCR and microarray analysis.

### Cycloheximide treatment

Cycloheximide (CHX) was dissolved in ethanol to make 20 mM stock solutions to be used in the experiments. Similar to the DEX treatment, 6‐d‐old seedlings of *GR:RSL4 rsl2 rsl4* plants were used for the CHX treatment. For CHX treatment, seedlings were flooded with 20 ml of MS liquid containing 20 μl of CHX (CHX treated). The seedlings were further treated with 20 μM CHX and 20 μM DEX (DEX+CHX treated) by flooding with 20 ml of MS liquid medium containing 20 μl of DEX and 20 μl of CHX. After treatment, the root tips were sampled at several time‐points for qRT‐PCR and microarray analysis.

### Microarray hybridization

Gene expression in root hairs of *GR:RSL4 rsl2 rsl4* seedlings under different treatments (mock, DEX, and DEX+CHX treated) were compared using the Affymetrix (Santa Clara, CA, USA) Gene Chip Arabidopsis ATH1 Genome Array. Total RNA was isolated from three independent biological replicates. The RNA samples from the root tips of the RSL4 inducible lines under different treatments were quantified using a NanoDrop ND1000 spectrophotometer (Thermo Fisher Scientific, USA) and assessed using an Agilent 2100 bioanalyzer (Agilent Technologies, Santa Clara, CA, USA). cDNA was synthesized from 4 μg of total RNA using one‐cycle target labelling and control reagents (Affymetrix) to produce biotin‐labelled cDNA. Following fragmentation, the cDNA was hybridized to the Arabidopsis ATH1 Genome Array. Each microarray was washed and stained with streptavidin‐phycoerythrin in a Fluidics station 450 (Affymetrix) and scanned at 1.56‐μm resolution in a GeneChipScanner 3000 7G System (Affymetrix). All the analyses, including the hybridization, staining, washing and screening for quality of the arrays, were carried out at the Genomics Service of the Centro Nacional de Biotecnología, Madrid, Spain (Consejo Superior de Investigaciones Científicas).

### Gene expression analysis

For background correction, normalization, and expression level summarization, the multi‐array analysis algorithm was used (Irizarry *et al*., 2003). Statistical analysis and graphical visualization of data were carried out using the interactive tool Fiesta http://bioinfogp.cnb.csic.es/tools/FIESTA/index.php). In order to determine changes in^ ^gene expression, an analysis of variance was used. *P*‐values were adjusted by false‐discovery rate to correct for multiple testing (Benjamini & Hochberg, 1995). Genes with adjusted *P*‐values of ≤ 0.001 were selected as statistically significant. They were considered to be differently expressed if the change in^ ^expression was > 2‐fold (up‐regulated). The values obtained for the DEX‐treated samples were compared with those obtained for mock‐treated samples to identify the genes induced by RSL4. Further, in order to determine if the gene expression of these RSL4‐induced genes are unaffected upon adding CHX, the values for the DEX+CHX‐treated samples were compared with those for the mock. The genes that exhibited similar values in both analyses were taken as putative direct target genes of RSL4.

### Root hair length measurements

To measure the length of root hairs in plants homozygous for T‐DNA insertions, root hairs located 3 mm from the root tip of 6‐ to 7‐d‐old seedlings were imaged under the Leica DFC310 FX camera mounted on a Leica M165 FC stereo microscope. The digital images obtained were used directly for root hair length measurement using imagej software https://imagej.nih.gov/ij/. Twenty seedlings of each genotype were measured.

## Results

**Figure 1:**
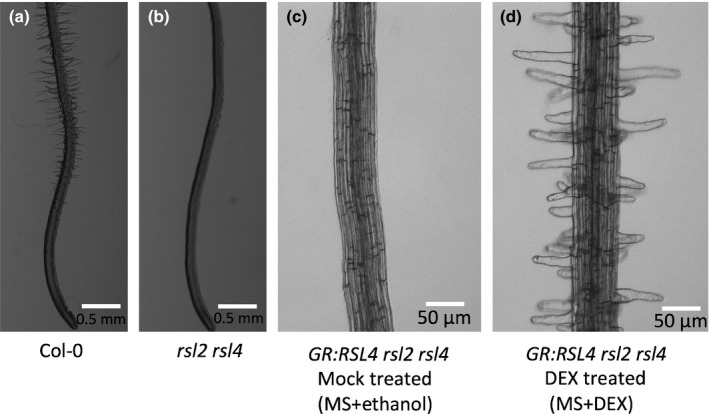
Dexamethasone (DEX) treatment induced root hair development in the *Arabidopsis thaliana Glucocorticoid Receptor (GR):ROOT HAIR DEFECTIVE SIX‐LIKE4* (*RSL4*) *rsl2 rsl4* background. (a, b) Root hair development in 6‐d‐old *Arabidopsis thaliana* Col‐0 and *rsl2 rsl4* seedlings. (c, d) *GR:RSL4 rsl2 rsl4* line 24* *h after mock (MS + ethanol) treatment (c) and after DEX treatment (d).

**Figure 2:**
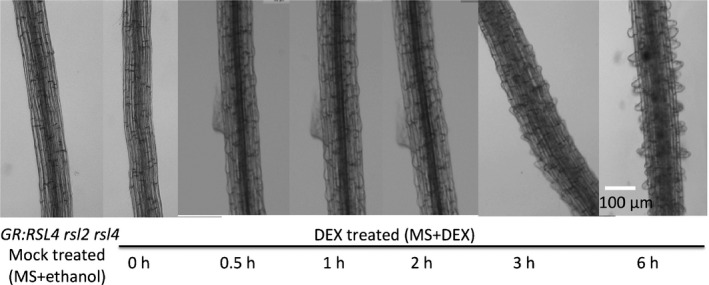
Dexamethasone (DEX) treatment induced root hair initiation in the *Arabidopsis thaliana Glucocorticoid Receptor (GR):ROOT HAIR DEFECTIVE SIX‐LIKE4* (*RSL4*) *rsl2 rsl4* background within 30 min. Images of root hair development in the *A. thaliana GR:RSL4 rsl2 rsl4* root at different times after DEX treatment are shown compared with 6 h after mock (Murashige and Skoog (MS) + ethanol) treatment.

The bHLH transcription factor RSL4 is necessary and sufficient for post‐mitotic cell growth in Arabidopsis root hairs (Yi *et al*., 2010). We hypothesized that the immediate targets of RSL4 would be required for root hair elongation. To identify downstream targets of RSL4, we developed an inducible system by expressing a glucocorticoid receptor:RSL4 fusion protein (GR:RSL4) in the root hairless *rsl2 rsl4* double mutant background (Lloyd *et al*., 1994). The GR‐RSL4 protein is located in the cytoplasm and moves to the nucleus upon exposure to a synthetic steroid, DEX. Once in the nucleus, GR:RSL4 will be active and promote the expression of RSL4‐regulated genes and induce root hair elongation. MS+ethanol‐treated (mock) *GR:RSL4 rsl2 rsl4* plants were root hairless (identical to *rsl2 rsl4* double mutants), indicating that the GR:RSL4 protein is not transcriptionally active when mock treated (Fig. 1a–c). Upon DEX treatment, the *GR:RSL4 rsl2 rsl4* seedlings developed root hairs (Fig. 1d), consistent with the induction of *RSL4* activity by DEX. Root hairs were visible as bulges emerging from the epidermal surface of trichoblasts within 30 min after DEX treatment (Fig. 2). This indicates that DEX‐mediated induction of GR‐RSL4 activity is rapid. Taken together, these data indicate that root hair development can be induced by DEX treatment in the *GR:RSL4 rsl2 rsl4* background.

**Figure 3:**
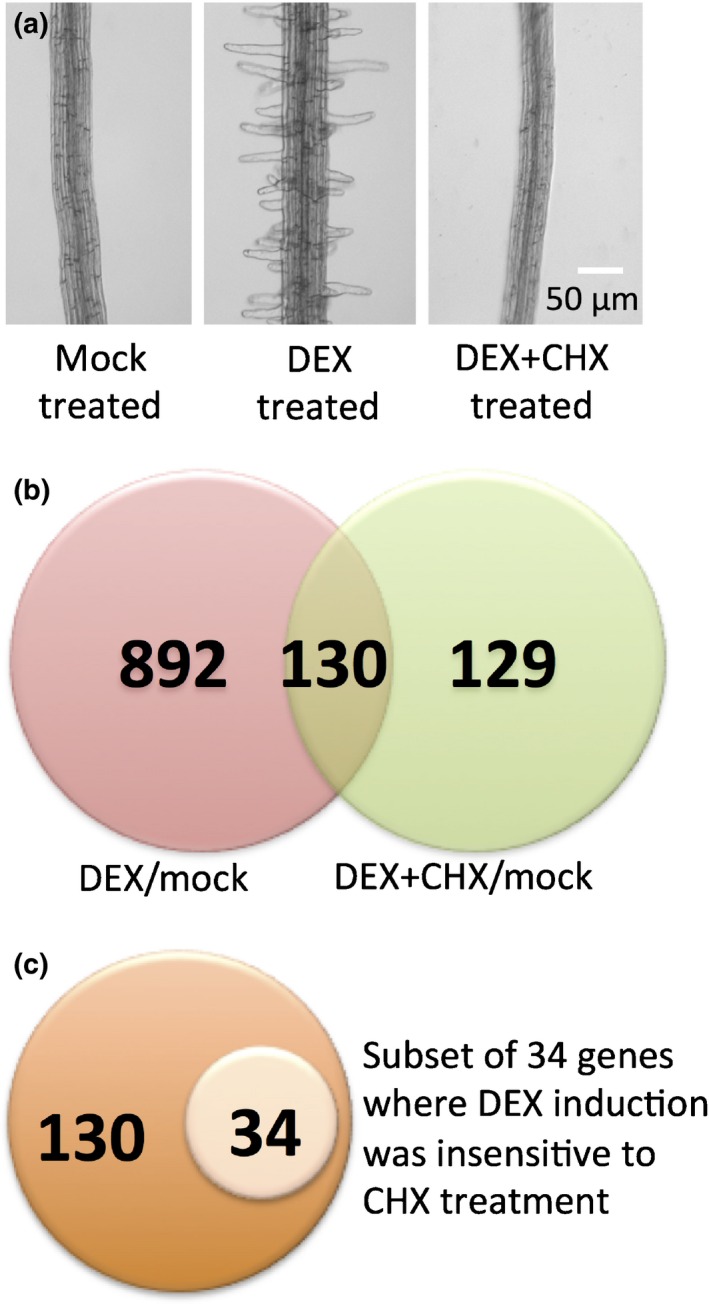
Identification of genes whose induction in the *Glucocorticoid Receptor (GR):ROOT HAIR DEFECTIVE SIX‐LIKE4* (*RSL4*) *rsl2 rsl4* background by dexamethasone (DEX) is insensitive to cycloheximide (CHX). (a) Development of root hairs 24 h after an *Arabidopsis thaliana GR:RSL4 rsl2 rsl4* plant was treated with Murashige and Skoog (MS) medium containing 20 μM DEX. No root hairs developed after treatment of the seedling with DEX + CHX and MS + ethanol (mock). (b) Venn diagram of genes induced upon RSL4 activation under different treatments. Pink and green circles represent genes induced only in the DEX/mock and DEX + CHX/mock sample, respectively. The brown region represents genes (130) that were induced significantly (*P *≤ 0.001) upon RSL4 activation both in DEX/mock and DEX + CHX/mock samples. (c) A subset of 34 genes of the 130 genes which exhibited a minimum two‐fold increase in expression upon DEX treatment and no change in expression levels upon addition of CHX were selected as putative direct targets of RSL4.

To identify the immediate RSL4 target genes that affect root hair growth, RSL4 activity was induced by DEX in the presence of the protein synthesis inhibitor CHX (Fig. 3). Only genes that do not depend on protein synthesis will be induced by the combined DEX and CHX treatment of *GR:RSL4 rsl2 rsl4* plants; that is, those genes that are regulated by the GR:RSL4 fusion protein directly. The development of root hairs was inhibited upon addition of CHX, indicating that CHX prevents hair development by inhibiting the translation of proteins required for root hair growth (Fig. 3a). However, as gene transcription is not inhibited by CHX, comparing the transcriptional profiles of roots treated with DEX with roots treated with both DEX and CHX can identify putative direct targets of RSL4. Genes that are induced both by DEX treatment and by the combined DEX and CHX treatment but whose levels of induction remain unaltered in the two treatments are putative direct RSL4 targets. RNA was isolated 6 h after mock treatment, DEX treatment and the combined DEX and CHX treatment of *GR:RSL4 rsl2 rsl4* roots. Steady‐state mRNA levels were measured using Affymetrix ATH1 arrays.

To identify genes whose expression was induced by DEX treatment, we selected genes whose expression was at least twice as high in the DEX‐ and DEX+CHX‐treated *GR:RSL4 rsl2 rsl4* roots compared with mock‐treated *GR:RSL4 rsl2 rsl4* roots. This identified 1022 genes whose expression was increased at least two‐fold by DEX treatment compared with mock treatment after applying a stringent filter, that is, *P*‐value < 0.0001 (Table S2). A total of 259 genes were more highly expressed in DEX+CHX compared with mock samples with *P*‐value < 0.001 (Table S3). The expression of 130 genes was significantly induced in both the DEX‐treated samples and the DEX+CHX‐treated samples; that is, 130 genes were common to both data sets (DEX/mock and DEX+CHX/mock) (Fig. 3b; Table S4). To identify the putative RSL4 direct target genes among this set of 130 genes, those DEX‐induced genes whose expression was insensitive to the addition of CHX were identified. The expression of 34 of the 130 genes (26% of the 130 genes selected) was identical in both DEX and DEX+CHX treatments (Fig. 3c). These 34 DEX‐inducible genes whose transcription was insensitive to CHX treatment were potential targets of RSL4 transcriptional regulation (Fig. 3c; Table 1).

**Table 1:** Thirty‐four *Arabidopsis thaliana* genes whose expression was more than twice as high in dexamethasone (DEX)‐treated *Glucocorticoid Receptor (GR):ROOT HAIR DEFECTIVE SIX‐LIKE4* (*RSL4*) *rsl2 rsl4* plants as in mock‐treated plants and whose DEX induction was insensitive to cycloheximide (CHX) treatment

Accession code	Gene symbol	Protein encoded/biological function
AT4G01120	*GBF2*	bZIP transcription factor that binds to the G‐box/blue light‐mediated development
AT5G02780	*GSTL1*	Glutathione transferase lambda 1/thiol transferase activity
AT1G21610	*UBN1*	Wound‐responsive family protein/response to wounding
AT2G27775		*Arabidopsis thaliana* unknown protein
AT5G58375		*Arabidopsis thaliana* unknown protein
AT5G43430	*ETFBETA*	Electron transfer flavoprotein beta/electron carrier activity
AT1G27360	*SPL11*	Squamosa promoter‐like 11/regulate timing of transition from vegetative to reproductive phase, transcription factor
AT3G56210		ARM repeat superfamily protein
AT4G27620		*Arabidopsis thaliana* unknown protein
AT5G03540	*EXO70A1*	Exocyst subunit EXO70 family protein A1/exocytosis
AT1G01800	*NADBP*	NAD(P)‐binding Rossmann‐fold superfamily protein/oxidoreductase activity
AT3G17090	*PP2C*	Protein phosphatase 2C family protein/dephosphorylation
AT3G18670		Ankyrin repeat family protein
AT1G26940	*CLP*	Cyclophilin‐like protein/protein folding, isomerization
AT1G35670	*CDPK2/CPK11*	Calcium‐dependent protein kinase/induced by drought and high salt, positive regulator of ABA signalling
AT3G27700		Zinc finger (CCCH‐type) protein/DNA, RNA binding
AT1G30870	*PER7*	Peroxidase superfamily protein/haem binding, metal ion binding, peroxidase activity
AT4G35890	*LARP 1C*	LAM domain‐containing protein/leaf senescence
AT3G24880	*EAF 1A*	Nuclear protein interacting with NuA4 histone acetyltransferase complex/DNA, protein binding
AT3G23540		Alpha/beta‐hydrolase superfamily protein
AT3G57570		ARM repeat superfamily protein
AT4G26400		RING/U‐box protein/zinc ion binding, response to chitin
AT2G22910	*NAGS1*	N‐acetyl‐l‐glutamate synthase 1/arginine biosynthesis
AT5G61570		Protein kinase family protein/phosphorylation
AT3G24350	*SYP32*	SYNTAXIN OF PLANTS 32/vesicle‐mediated transport
AT4G00830	*LIF2*	LHP1‐Interacting Factor 2, RNA‐binding partner of LHP1 (Polycomb complex component)/controls flowering, cell fate
AT1G75850	*VPS35B/LAZ4*	Vacuolar protein sorting‐associated protein 35/intracellular protein transport
AT5G65205		NAD(P)‐binding Rossmann‐fold superfamily protein, oxidoreductase activity
AT4G26240		*Arabidopsis thaliana* unknown protein
AT5G05987	*PRA1.A2*	PRENYLATED RAB ACCEPTOR 1.A2, vesicular transport
AT2G25570		*Arabidopsis thaliana* unknown protein
AT2G17670		Tetratricopeptide repeat (TPR)‐like superfamily protein
AT5G59870	*H2A.W.6/HTA6*	Histone H2A protein/chromatin binding, heterochromatin organization
AT1G22620	*SAC1*	SUPPRESSOR OF ACTIN (SAC) domain containing phosphatidylinositol‐4,5‐bisphosphate 5‐phosphatase/morphogenesis, cell wall synthesis, and actin organization

EAF 1A, ESA1‐associated factor 1A; GBF2, G‐Box binding factor 2; LARP 1C, La‐related protein 1C; UBN1, Ubinuclein 1.

**Figure 4:**
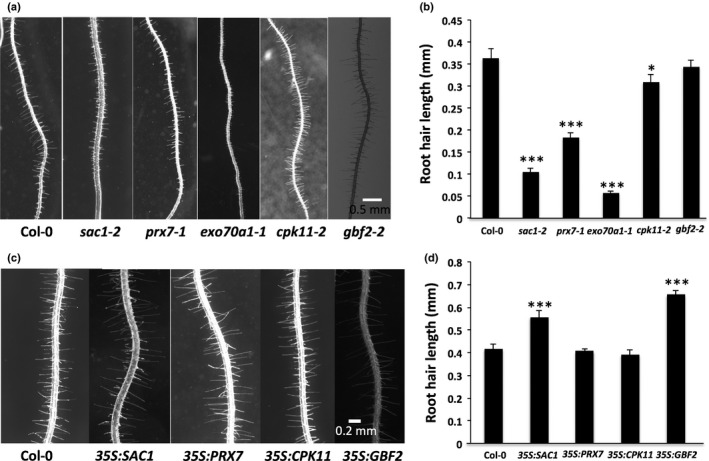
Targets of ROOT HAIR DEFECTIVE SIX‐LIKE4 (RSL4) regulation encode proteins involved in cell signalling, cell wall modification and secretion that are required root hair growth. (a) Seven‐day‐old *Arabidopsis thaliana* wild‐type (Col‐0 roots), *suppressor of actin 1‐2* (*sac1‐2*), *peroxidase7‐1* (*prx7‐1*), *exocsyt subunit 70a1‐1* (*exo70a1‐1)*,*calcium‐dependent protein kinase11‐2* (*cpk11‐2*) and *g‐box binding factor2‐2* (*gbf2‐2*) roots. (b) Root hair length measurements of 7‐d‐old wild‐type (Col‐0 roots), *sac1‐2, prx7‐1, exo70a1‐1*,* cpk11‐2* and *gbf2‐2* roots (*n *=* *15). (c) Images of 7‐d‐old roots of wild‐type (Col‐0) and plants transformed with *35S:SAC1, 35S:PRX7, 35S:CPK11* and *35S:GBF2*; (d) root hair length measurements of wild‐type (Col‐0) and plants transformed with *35S:SAC1, 35S:PRX7, 35S:CPK11* and *35S:GBF2* (*n *=* *15). Error bars represent SEM. *, *P *<* *0.05; ***, *P *<* *0.001 by Student's *t*‐test.

Genes whose expression is induced by DEX treatment in a CHX‐independent manner in *rsl2 rsl4 GR:RSL4* plants encode proteins involved in a variety of cellular activities, including signalling, secretion and cell wall modification (Table 1). As RSL4 is a key regulator of root hair development, we predicted that at least some of these genes would be required for root hair elongation. We determined the root hair phenotype in plants homozygous for T‐DNA insertions in nine of the 34 putative RSL4 target genes. These nine genes were expressed at high levels in the root hair and/or other root tissues as reported in the Arabidopsis e‐FP Browser (Table S5) (Winter *et al*., 2007). They included *G‐BOX BINDING FACTOR2* (*GBF2*) (AT4G01120), *EXOCSYT SUBUNIT 70A1* (*EXO70A1*) (AT5G03540), *NAD(P) BINDING PROTEIN* (*NADBP*) (AT1G01800), *PROTEIN PHOSPHATASE 2C FAMILY PROTEIN (PP2C)* (AT3G17090), *CYCLOPHILIN‐LIKE PROTEIN* (*CLP*) (AT1G26940), *CALCIUM‐DEPENDENT PROTEIN KINASE11* (*CPK11/CDPK2*) (AT1G35670), *PEROXIDASE7* (*PRX7*) (AT1G30870), *VACUOLAR PROTEIN SORTING‐ASSOCIATED PROTEIN 35B* (*VPS35B/LAZ4*) (AT1G75850) and *SUPPRESSOR OF ACTIN* (*SAC1*) (AT1G22620). Plants with reduced function of four of these nine genes developed shorter root hairs than the wild‐type; root hair initiation was normal but the subsequent elongation by tip growth was defective (Fig. 4a,b). No difference was observed in root hair length between the wild‐type and plants homozygous for T‐DNA insertions in the remaining five genes (Figs 4a,b, S1).

*SAC1* (*AT1G22620*) encodes a phosphatidylinositol (PI) phosphatase that dephosphorylates phosphotidylinositol 3,5 bisphosphate to form phosphotidylinositol 3‐phosphate (Zhong *et al*., 2005). While the root hairs initiated normally on plants that were homozygous for T‐DNA insertions in *SAC1*, root hair elongation was defective (Fig. 4a); wild‐type root hairs were 0.36 mm (± 0.02) (mean ± SEM) long while those root hairs that elongated in *sac1* were 0.10 mm (± 0.01) (mean ± SEM) long (Fig. 4b). This suggests that SAC1 is required for the initiation of tip growth. As *SAC1* is required for root hair elongation, we hypothesized that constitutive expression of *SAC1* would lead to the formation of longer hairs compared with the wild‐type. To test this hypothesis, we constitutively expressed *SAC1* by transforming wild‐type plants with a cauliflower mosaic virus (*CaMV*) *35S:SAC1* construct (Fig. 4c,d). Root hairs were 0.42 mm (± 0.02) (mean ± SEM) long in the wild‐type while root hairs were 0.56 mm (± 0.02) (mean ± SEM) long in *35S:SAC1* transformed lines (Fig. 4d). This indicates that not only is SAC1 required for root hair growth but that its activity is limiting for growth. As auxin induces RSL4 activity, we tested the effect of auxin on the expression of *SAC1*. We found that the mRNA levels of SAC1 were increased two‐fold upon 100 nM IAA treatment compared with controls (Fig. S2). These data are consistent with the hypothesis that auxin positively regulates *SAC1* expression via RSL4.

*PRX7* encodes a class III peroxidase that plays a role in the cleavage and crosslinking of the cell wall polysaccharides (Tognolli *et al*., 2002; Valerio *et al*., 2004). The root hairs of plants homozygous for T‐DNA insertions into *PRX7* are almost half the length of wild‐type root hairs (Fig. 4a); *prx7‐1* root hairs were 0.18 mm (± 0.01 mm) (mean ± SEM) and wild‐type root hairs were 0.36 mm (± 0.02) (mean ± SEM) (Fig. 4b). Root hair length was indistinguishable between the wild‐type and wild‐type plants transformed with the *CaMV 35S:PRX7* construct (Fig. 4c,d). This indicates that PRX7 is required for root hair elongation but that increased levels of *PRX7* expression are not sufficient for hair elongation.

*EXO70A1* is a subunit of exocyst, a secretory vesicle tethering complex that spatially regulates exocytosis into the plasma membranes of growing cells (Li *et al*., 2010). Trichoblasts of plants homozygous for T‐DNA insertion in *EXO70A1, exo70a1‐1*, develop swellings that do not elongate, indicating a defect in the transition to tip growth (Fig. 4a); wild‐type root hairs were 0.36 ± 0.02 mm (± SEM) long whereas *exo70a1‐1* mutant root hairs were 0.06 ± 0.01 mm in length (Fig. 4b). A similar root hair growth defect was seen in plants with another mutant allele, *exo70a1‐2* (Fig. S3). Both these alleles have been previously reported to confer defects in root hair growth (Synek *et al*., 2006). The phenotypes of *exo70a1* mutant root hairs suggest that *EXO70A1* activity is required for the initiation of tip growth from the bulge that forms at the earliest stage of root hair development.

Calcium‐dependent protein kinases (CPKs) catalyse Ca^2+^‐dependent phosphorylation in cell signalling processes (Patil *et al*., 1995; Sanders *et al*., 1999). *CPK11* was identified as a putative direct downstream target of RSL4. Loss‐of‐function *cpk11‐2* mutants developed shorter root hairs than Col‐0 (Fig. 4a). *cpk11‐2* root hairs were 0.31 mm ± 0.01 mm (mean ± SEM) long, compared with 0.36 mm ± 0.02 (mean ± SEM) in Col‐0 (Fig. 4b). To determine if overexpression of *CPK11* increased root hair elongation, we transformed wild‐type plants with the *35S:CPK11* construct. Root hair length in plants transformed with *35S:CPK11* was identical to that of untransformed controls (Fig. 4c,d), indicating that the levels of *CPK11* expression in wild‐type are not limiting for growth. Maintenance of a tip‐focused cytoplasmic calcium (Ca^2+^) gradient is indispensable for root hair growth and *CPK11* may be a component of the calcium signalling pathway, regulating RSL4‐mediated root hair elongation.

One of the 34 putative targets of RSL4 encoded a protein with a putative role in transcription. *GBF2* was induced by DEX in a CHX‐insensitive fashion by *RSL4:GR*. GBF2 has been reported to control blue light‐mediated growth and development in Arabidopsis. To determine if GBF2 is involved in root hair elongation, we generated loss‐of‐function and gain‐of‐function *GBF2* lines. Root hair development in plants homozygous for a T‐DNA insertion in *GBF2* (*gbf2*‐*2*) is identical to that of the wild‐type (Fig. 4a,b). However, root hairs of plants transformed with *CaMV 35S:GBF2* were longer (0.66 ± 0.02 mm (SEM)) than those of the wild‐type (0.41 ± 0.02 mm (SEM)) (Fig. 4c,d). This indicates that RSL4‐regulated *GBF2* expression promotes root hair elongation but it is likely that another gene can compensate for reduced *GBF2* function.

Loss of function of four genes had no impact on root hair elongation; the length of *nadbp‐1, pp2c‐1*,* clp‐1*,* vps35a‐1* mutant root hairs was identical to that of the wild‐type (Fig. S1). To identify a function in root hair elongation in the four genes for which loss‐of‐function mutants provided no functional information, we generated gain‐of‐function lines. Root hair length was indistinguishable from that of wild‐type controls (0.41 ± 0.02 mm (SEM)) in plants transformed with *CaMV 35S:NADBP, 35S:PP2C*,* 35S:CLP* and *35S:VPS35B* constructs. This indicates that overexpression of none of these four genes is sufficient for root hair elongation. Therefore, we cannot define a role for these genes in root hair development. It is possible that these genes are not involved in root hair elongation, that genetic redundancy compensates for their loss of function or that these genes are not required for root hair elongation in the conditions used in this analysis.

Taken together, these results suggest that genes regulating secretion, cell wall modifications and signal transduction modulate RSL4‐mediated root hair growth.

## Discussion

We have shown that RSL4 controls root hair elongation by regulating the transcription of genes that are required for cell growth. These include genes encoding proteins involved in vesicle trafficking, lipid signalling and those encoding RAB GTPase regulators among others. There are also genes that encode proteins involved in cellular activities associated with transcriptional regulation and chromatin components among the 34 putative targets. Four genes (12%) encode proteins of unknown function in plants and their function remains to be determined.

Among the genes required for root hair growth that are regulated by RSL4 is *SAC1*. While loss of SAC1 function results in short root hairs, gain of function results in longer root hairs than those of the wild‐type. Together, these gain‐of‐function and loss‐of‐function data are strong evidence that SAC1 is required for elongation during root hair cell morphogenesis. *fragile fibre7* (*fra7*) mutants were identified because they have defects in the morphogenesis of fibre cells and the defect is caused by mutations in the *SAC1* gene (Zhong *et al*., 2005). This indicates that FRA7/SAC1 is required for morphogenesis of diverse cell types in the plant. *SAC1* encodes a PI phosphatase. PIs regulate several cellular processes such as signal transduction, vesicle trafficking and the organization of the actin cytoskeleton (Zhong *et al*., 2005). SAC domain‐containing phosphatases are a group of enzymes that were first identified in yeast and animals and have been shown to hydrolyse phosphates on several positions of the inositol head group of PI (Hughes *et al*., 2000). There are nine SAC domain‐containing PI phosphatase‐like proteins (SAC1−9) in Arabidopsis (Zhong & Ye, 2003). Mutations in the *SAC7/ROOT HAIR DEFECTIVE H4 (RHD4)* gene result in the formation of short, bulged and branched root hairs (Thole *et al*., 2008). Together, these data indicate that members of the SAC family of phosphatases are involved in root hair elongation.

The defective root hair elongation in the *sac1‐2* mutant indicates that SAC1‐mediated phosphorylation of phosphotidylinositol phosphates (PIPs) is important for root hair growth. There is evidence that other proteins that catalyse the phosphorylation or dephosphorylation of PIs are also required for root hair elongation. It is hypothesized that SAC1 dephosphorylates phosphotidylinositol 3,5‐bisphosphate(PtdIns) (3,5)P2 to phosphatidylinositol 3‐phosphate PtdIns(3)P (Zhong *et al*., 2005). The reverse reaction is catalysed by a PtdIns3P 5‐kinase (*FORMATION OF APLOID AND BINUCLEATE CELLS, FAB1*), to form PtdIns(3,5)P2. Both *FAB1* gain‐of‐function (overexpression) and loss‐of‐function mutants develop short root hairs indicative of the role of *FAB1* in root hair development (Hirano *et al*., 2011). Other kinase enzymes that phosphorylate PI substrates have been implicated in root hair development. For example, VPS34 is a class III PI3‐kinase that phosphorylates PI to form PtdIns(3)P. Inhibition of VPS34 activity results in the development of shorter root hairs with slower growth rates than wild‐type (Lee *et al*., 2008). Taken together, these results demonstrate the importance of the PtdIns(3)P pathway in regulating root hair cell growth. RSL4‐regualted *SAC1* transcription is a key element in the regulation of the pathway.

Secretion is necessary for cell growth; it delivers lipids, polysaccharides and proteins that constitute the new plasma membrane and cell wall of the growing cell. We report that the transcript levels of *EXO70A1* are probably directly regulated by the bHLH transcription factor RSL4. *EXO70A1* encodes a subunit of the exocyst protein complex (Synek *et al*., 2006). This complex regulates the first contact (tethering) between secretory vesicles and the target membrane. It plays a crucial role in the transport and secretion of new wall and membrane materials from the endo membrane system to the growing surface. Kulich *et al*. (2010) showed that *EXO70A1* is essential for the polarized delivery of cell wall pectins during seed coat development (Kulich *et al*., 2010). EXO70A1 is also involved in the polar recycling of PIN1 and PIN2 and thus participates in polar auxin transport (Drdova *et al*., 2013). It has previously been reported that root hair elongation is defective in *exo70a1* mutants with reduced function (Synek *et al*., 2006). *exo70a1* mutants developed bulges from which root hairs did not elongate, suggesting that there is a defect in transition to tip growth in this background. The defective root hair elongation phenotype of *exo70a1‐2* mutants is partially rescued by Napthalene Acetic Acid (NAA) treatments (Synek *et al*., 2006). The inability of NAA to completely rescue root hair elongation in this mutant indicates that auxin‐modulated root hair elongation acts least partially through *EXO70A1*. The partial rescue of the Exo70a1 mutant phenotype is probably attributable to auxin regulation of other activities that in part compensate for the loss of *EXO70A1* function. In rice, *OsEXO70A1* has been implicated in the regulation of nutrient assimilation which may be attributable to defects in root hair‐mediated nutrient uptake (Tu *et al*., 2015). Given that RSL4 activity is increased by low nutrient availability which induces root hair elongation, our data are consistent with a model in which RSL4‐mediated *EXO70A1* transcription is required for tip growth.

Root hair growth is accompanied by the modification of the plant cell wall and our finding that *PRX7*, a class III peroxidase gene, is regulated by RSL4 suggests that RSL4 promotes root hair growth, in part by regulating PRX7‐modulated cell wall modification. Plant cell walls are dynamic structures that determine shape and mechanically support the cell. Several enzymes including class III peroxidases have been implicated in the cleavage and reassembly of the cell wall components during dynamic transformations. There are reports that peroxidases enable wall loosening by generating reactive oxygen species (ROS) (Passardi *et al*., 2004). *PRX33* and *PRX34* have been shown to be involved in root elongation by promoting cell wall loosening (Passardi *et al*., 2006). Mutations in two peroxidase genes, *PRX44* and *PRX57*, resulted in root hair cell wall rupture indicative of defective cell walls at the growth tip where walls are thin and mechanically weakest (Kwon *et al*., 2015). Our finding that PRX7 acts downstream of RSL4 indicates that cell wall modifications play an important role in the RSL4‐mediated root hair elongation process.

We identified *CPK11* as a potential direct target of RSL4 using an inducible RSL4 protein driven from its native promoter. CPK11 encodes a calcium‐dependent protein kinase that has been shown to regulate the abscisic acid signalling pathways in Arabidopsis (Zhu *et al*., 2007). CPKs are characterized by the presence of a C‐terminal calmodulin‐like regulatory domain with four calcium‐binding EF hands and their activity is regulated by Ca^2+^ signals (Patil *et al*., 1995; Sanders *et al*., 1999). *Medicago truncatula* CDPK1 has been shown to regulate root hair elongation. Silencing of *CDPK1* by RNA interference resulted in the development of short root hairs (Ivashuta *et al*., 2005). Furthermore, the maintenance of tip‐focused cytoplasmic calcium (Ca^2+^) is indispensable for root hair growth. A local elevation in the concentration of cytoplasmic Ca^2+^ in the growing tip is required for root hair elongation (Bibikova *et al*., 1997). *CPK11* may be a component of the calcium signalling pathway involved in RSL4‐mediated root hair elongation.

Five genes did not mutate to a root hair defective phenotype. This suggests that they are not involved in root hair elongation. However, it is possible that similar genes have overlapping functions and functionally compensate in the single mutants that we characterized here. Such genetic redundancy is thought to be common and the observation that there are many closely related genes in families supports this hypothesis. Genetic redundancy can be determined by generating lines in which there are mutations in more than one gene. It is also formally possible these genes are not involved in root hair growth. These genes may carry out other roles in the root hair that are not related to growth per se but some other function that is restricted to root hair cells, as RSL4 expression is restricted to this cell type.

## Author contributions

P.V. carried out the transcriptional analysis under the supervision of S.D. P.V. and S.D. analysed the data of the transcriptional analysis. P.V., S.D. and L.D. analysed the complete data set of the study. L.D. conceived and managed the project. S.D. and L.D. wrote the paper.

## Supporting information

Please note: Wiley Blackwell are not responsible for the content or functionality of any supporting information supplied by the authors. Any queries (other than missing material) should be directed to the *New Phytologist* Central Office.

**Fig. S1** Six‐day‐old *Arabidopsis thaliana* wild‐type Col‐0, *nadbp‐1, pp2c‐1, clp‐1* and *vps35a‐1* roots.
**Fig. S2** *SAC1* mRNA level in the absence and presence of exogenously applied 100 nM IAA.
**Fig. S3** Six‐day‐old *Arabidopsis thaliana* wild‐type (Col‐0) and *exo70a1‐2* mutants with short root hairs.
**Table S1** List of primers used in this study
**Table S5** List of *Arabidopsis thaliana* mutants used in this study with details of T‐DNA insertions

**Table S2** 1022 genes in *Arabidopsis thaliana* whose expression was more than twice as high in DEX‐treated *GR:RSL4 rsl2 rsl4* plants as in mock‐treated plants with a *P*‐value < 0.0001

**Table S3** 259 genes in *Arabidopsis thaliana* whose expression was more than twice as high in DEX+CHX‐treated *GR:RSL4 rsl2 rsl4* as in mock‐treated plants with a *P*‐value < 0.001

**Table S4** 130 genes in *Arabidopsis thaliana* whose expression was increased by more than two‐fold in both DEX‐ and DEX+CHX‐treated *GR:RSL4 rsl2 rsl4* plants compared with mock treatment
