# Supplementary material for: ROOT HAIR DEFECTIVE SIX‐LIKE4 (RSL4) promotes root hair elongation by transcriptionally regulating the expression of genes required for cell growth
Source: New Phytol. 2016 Jul 25;212(4):944–53. doi: 10.1111/nph.14095 (PMC5111604; doi:10.1111/nph.14095)
Supplement: Supplementary file 1 — Fig. S1 Six‐day‐old Arabidopsis thaliana wild‐type Col‐0, nadbp‐1, pp2c‐1, clp‐1 and vps35a‐1 roots. Fig. S2 SAC1 mRNA level in the absence and presence of exogenously applied 100 nM IAA. Fig. S3 Six‐day‐old Arabidopsis thaliana wild‐type (Col‐0) and exo70a1‐2 mutants with short root hairs. Table S1 List of primers used in this study Table S5 List of Arabidopsis thaliana mutants used in this study with details of T‐DNA insertions [file NPH-212-944-s001.pdf]

New Phytologist

***New Phytologist* Supporting Information**

Article title: **RSL4 promotes root hair elongation by transcriptionally regulating the expression of genes required for cell growth**

Authors: Priya Vijayakumar, Sourav Datta and Liam Dolan

Article acceptance date: 5 June 2016

The following Supporting Information is available for this article:

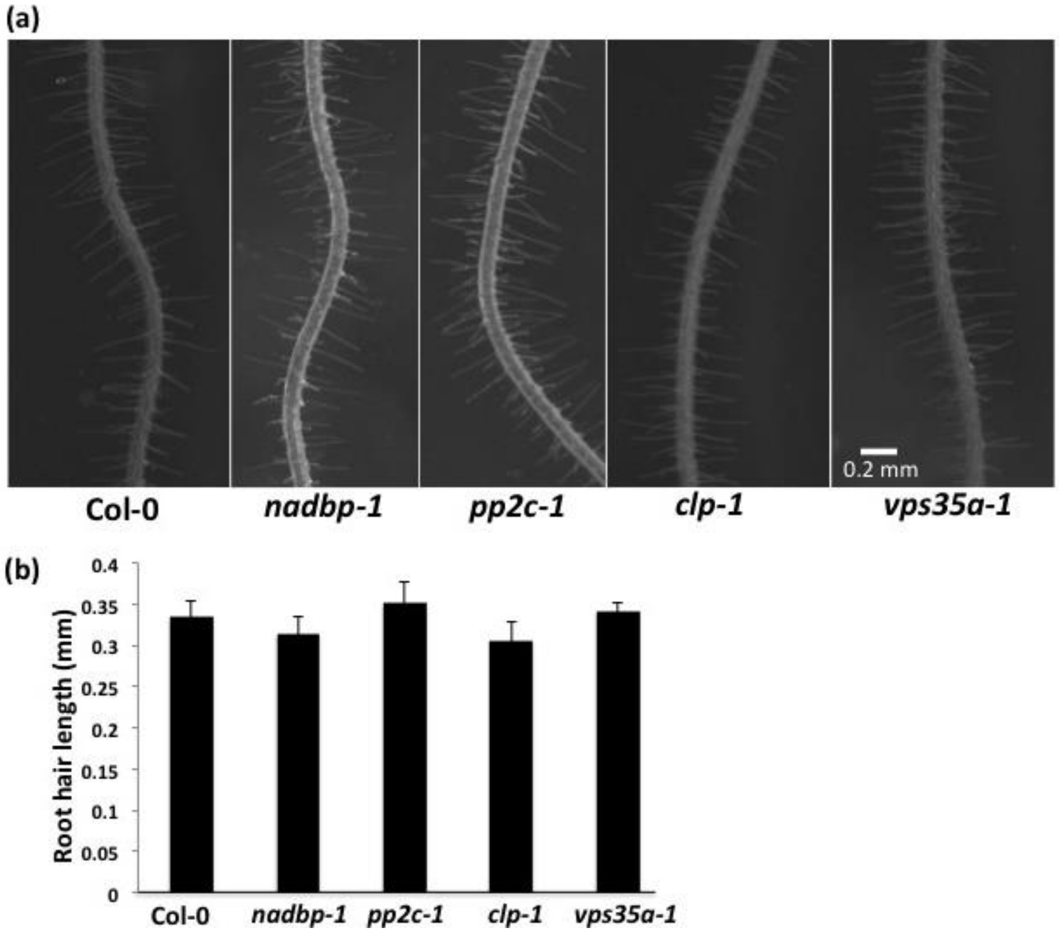

Fig. S1 (a) Images of 6-d-old *Arabidopsis thaliana* wild type Col-0, *nadbp-1, pp2c-1, clp-1* and *vps35a-1* roots grown on MS plates with 0.5% Phytagel. (b) Root hair length measurements of 6-d-old wild type (Col-0), *nadbp-1, pp2c-1, clp-1* and *vps35a-1* plants (*n* = 15). Error bars represent SEM.

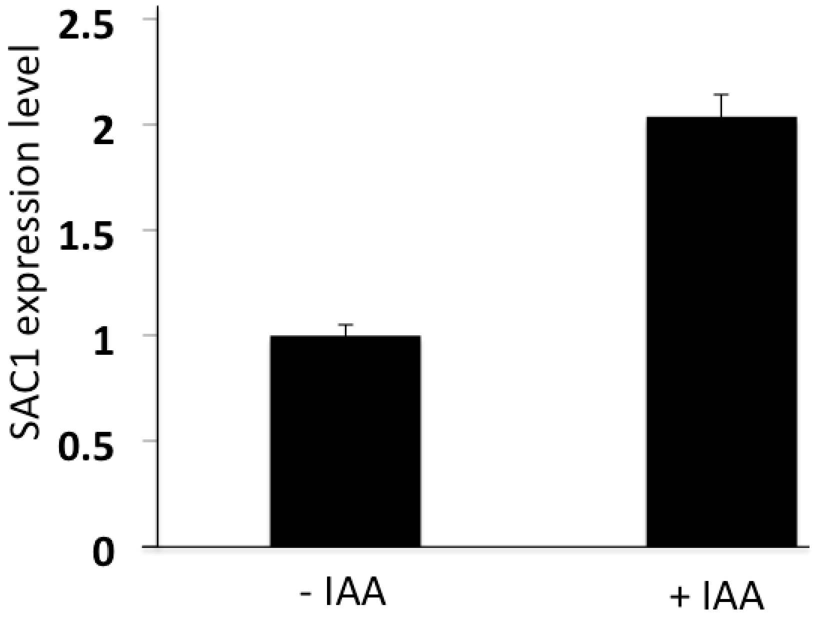

Fig. S2 *SAC1* mRNA level in absence and presence of exogenously applied 100 nM IAA (*n* = 3). Error bars represent SEM.

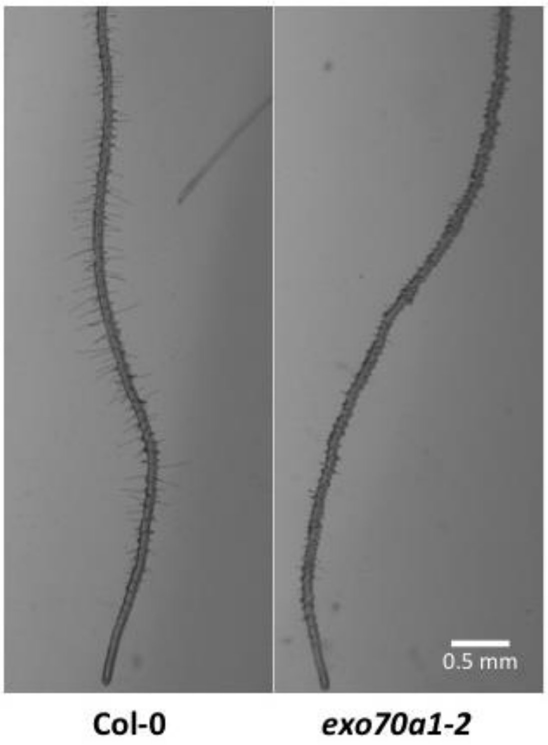

Fig. S3 Images of 6-d-old *Arabidopsis thaliana* wild type (Col-0) and *exo70a1-2* mutants with short root hairs grown on MS plates with 0.5% Phytagel.

Table S1 List of primers used in this study.

Primers used to confirm the GR: RSL4 fusion construct

Primers within the 5'UTR and promoter of *RSL4*

ATTB4: GGGGACAACTTTGTATAGAAAAGTTGATACGCGTTGGGCTTAAATG
ANTI578: CCTAAAACCTAAACGCGGTG

Primers within the CDS+3'UTR of *RSL4*

RDL2 KpnI: CGGGGTACCATGGACGTTTTTGTTGATGGT
RDL2 BamHI: CTTTTGTCTCGGCTTATGTGAGGATCCGCG

Primers within the GR sequence:

GR forward: CAAAGGGATTCAGCAAGCCAC
GR reverse: CCTTACCTACTGCTTCCAGACATTT

Table S2 1022 genes in *Arabidopsis thaliana* that were expressed more than twice as high in DEX treated *GR:RSL4 rsl2 rsl4* plants than in mock treated plants with a *P* value < 0.0001. (See separate Excel file.)

Table S3 259 genes in *Arabidopsis thaliana* that were expressed more than twice as high in DEX+CHX treated *GR:RSL4 rsl2 rsl4* than in mock treated plants with a *P* value < 0.001. (See separate Excel file.)

Table S4 130 genes in *Arabidopsis thaliana* whose expression was increased by more than two-fold in both DEX and DEX+CHX treated *GR:RSL4 rsl2 rsl4* plants compared to mock treatment. (See separate Excel file.)

New Phytologist

Table S5 List of mutants used in this study with details of T-DNA insertions

Mutant alleles	Gene name	Gene code	Insertion code
gbf2-2	GBF2	AT4G01120	SALK_206654
exo70a1-1, exo70a1-2	EXO70A1	AT5G03540	SALK_014826, SALK_135462
nadbp-1	NADBP	AT1G01800	SALK_009967
pp2c-1	PP2C	AT3G17090	SALK_203806
clp-1	CLP	AT1G26940	SALK_054035
cpk11-2	CDPK2/CPK11	AT1G35670	SALK_054495
prx7-1	PRX7	AT1G30870	GK_476A09-019131
vps35a-1	VPS35B/ LAZ4	AT1G75850	SALK_014345
sac1-2	SAC1	AT1G22620	SALK_070875
